# Assessment of General Practitioners’ Knowledge, Needs, and Barriers in Primary Healthcare Delivery in Emirates Health Services, United Arab Emirates: A Cross-Sectional Study

**DOI:** 10.7759/cureus.107196

**Published:** 2026-04-16

**Authors:** Fatima A Almeleh, Zainab M Alameeri, Sultana J Alzaabi, Asma S Alameeri, Hiba Y Alhammadi, Kareemah Alraeesi

**Affiliations:** 1 Primary Healthcare Department, Emirates Health Services, Dubai, ARE

**Keywords:** attitudes, continuing medical education (cme), general practitioners, knowledge, practices, primary healthcare

## Abstract

Background

General Practitioners (GPs) are central to primary healthcare delivery, yet data on their knowledge and continuing education needs within Emirates Health Services (EHS) are limited. This study aimed to assess GPs’ knowledge, needs, and barriers among GPs in EHS primary health centers in the United Arab Emirates.

Methods

A multi-center cross-sectional study was conducted between April and June 2025 across 63 EHS primary healthcare centers. Data were collected using a structured, self-administered questionnaire covering demographics, knowledge domains (common, chronic, and women’s and children’s health conditions), training needs, and perceived barriers. Knowledge levels were categorized using modified Bloom’s cut-offs. Descriptive statistics were reported as frequencies and percentages, and inferential analyses were performed using χ² tests, Fisher’s exact tests, t-tests, and one-way ANOVA, with statistical significance set at p < 0.05.

Results

Among the 127 participants, 55.9% (n = 71) were male, 36.2% (n = 46) were aged 41-50 years, and 40.9% (n = 52) had five to 10 years of experience. A high level of knowledge regarding common primary healthcare conditions was reported by 86.6% (n = 110) of GPs, and 63.8% (n = 81) showed high knowledge of chronic conditions, with significant associations between knowledge and both age (p = 0.013) and experience (p = 0.021). Knowledge of child and women’s health conditions was moderate to high in 78.7% (n = 100) of participants, with no significant demographic associations (p > 0.05). Regarding training needs, 49.6% (n = 63) indicated a moderate need and 43.3% (n = 55) a high need. Most respondents (63.0%, n = 80) stated that previous EHS training sessions had significantly improved their clinical skills.

Conclusion

GPs under EHS demonstrated generally high knowledge levels yet expressed substantial needs for continued professional training. Major barriers to development were time constraints and limited institutional support.

## Introduction

The World Health Organization (WHO) describes primary healthcare as an inclusive, community-centered approach that promotes health, prevents disease, and provides treatment and support close to where people live [1]. General Practitioners (GPs), who enter the medical workforce after graduating from medical school, often without pursuing a specialized degree, form the backbone of many healthcare systems worldwide [2]. As frontline healthcare providers, GPs deliver first-contact, continuous, and comprehensive care to individuals and families. Their responsibilities range from managing acute and chronic conditions to providing preventive services and health education [3]. Therefore, the sustainability and effectiveness of primary healthcare systems are highly dependent on the capacity and performance of GPs. Over time, the expectations placed on GPs have grown increasingly complex. While managing large patient volumes and limited consultation times, they are also expected to stay updated with evolving clinical guidelines, diagnostic advancements, treatment innovations, and the integration of digital health technologies [4]. For example, Andersen et al. identified that GPs managing chronic diseases like type 2 diabetes require layered information, including up-to-date guidelines, medication management strategies, and knowledge of potential complications [5].

Understanding GPs’ knowledge base, training needs, and perceived barriers is essential for strengthening PHC. Research in this area can inform the development of targeted professional development programs, optimize policy decisions and resource allocations, and ultimately improve the quality of care at the primary level. In the context of Emirates Health Services (EHS), a major governmental healthcare provider in the United Arab Emirates (UAE), GPs serve as the first point of contact for a diverse and rapidly growing population [6]. Assessing their preparedness to meet evolving healthcare demands is therefore critical. However, GPs’ effectiveness in such roles is often questioned due to the multifaceted and evolving nature of primary care, which demands multidisciplinary knowledge, diagnostic precision, and strong patient-centered communication skills [7]. A study in Pakistan found that non-specialist GPs reported inadequate understanding of chronic disease management, preventive care, and diagnostic protocols, particularly when compared to their specialist counterparts [8]. These deficiencies are especially concerning given the high burden of non-communicable diseases in the Gulf region.

Continuing Professional Development (CPD) is considered a crucial mechanism for addressing these knowledge gaps. However, access to CPD is not always equitable, convenient, or tailored to the unique needs of GPs without specialized training [9]. In the UAE, while no study has specifically stratified GPs by knowledge level, research in the Al Ain region revealed that many GPs, particularly those without board certification, struggled with implementing Clinical Practice Guidelines (CPGs), citing knowledge deficits and uncertainty about guideline applicability to their patient populations [10]. Globally, GPs seek relevant, practical, and easily accessible education that reflects the realities of their clinical environments. Strengthening primary care requires addressing personal, institutional, and systemic barriers through focused continuing medical education (CME) efforts, supportive policies, and the development of localized, context-sensitive guidelines. However, heavy workloads, limited access to resources, and lack of protected time for study remain major impediments [11]. Organizational barriers also play a significant role. GPs’ ability to practice evidence-based medicine (EBM) is hindered by time constraints, insufficient institutional support, and restricted access to clinical information due to paywalls or inadequate infrastructure [5]. These issues are particularly acute for solo practitioners and those in rural areas. Systemic factors, such as conflicting guidelines and a lack of leadership, further reduce the uptake of CPGs [7,8].

In the UAE, structural and systemic barriers also affect communication within PHC settings. A cross-sectional study across 12 primary healthcare centers (PHCs) in Dubai found time constraints to be the most significant barrier to effective communication. About 23.4% of patients reported experiencing time-related issues during consultations, and 50.6% of GPs identified time pressure as a moderate to very high risk to communication. Additional challenges included preoccupation with electronic medical records, the need to address multiple concerns in a single visit, and difficulty confirming patient comprehension, all of which stem from broader organizational inefficiencies [12]. While the UAE has invested in assessing physician performance in general, the literature has not adequately distinguished between board-certified family physicians and GPs who enter practice immediately after graduation. This lack of distinction limits our understanding of the specific educational and infrastructural needs of this cohort. By identifying knowledge gaps, training priorities, and both personal and institutional barriers, this study aims to support the development of practical, evidence-based strategies to empower GPs and enhance primary healthcare outcomes.

## Materials and methods

Study design and setting

This was a multi-center, cross-sectional study conducted between April and June 2025 across 63 PHCs operated under EHS. The study was designed to evaluate GPs’ knowledge, training needs, and perceived barriers in primary healthcare delivery.

Study participants

The study population included all 161 GPs employed in PHCs under EHS. Eligibility criteria required participants to be currently practicing within EHS PHCs, willing to participate, and able to provide informed consent. Exclusion criteria included GPs not affiliated with EHS, those unwilling to consent, or those who did not complete the questionnaire.

Sample size was calculated using the finite population correction formula for cross-sectional studies, assuming a total population of 161 GPs working in EHS PHCs, a 95% confidence level, a 5% margin of error, and an assumed population proportion of 0.5 to ensure maximal variability [13]. The minimum required sample size was calculated to be 114 participants. After adjusting for an anticipated 10% non-response, the final target sample was 125 participants.

Data collection instrument

Data were collected using a structured, self-administered questionnaire specifically developed for this study, consisting of 21 closed-ended items and one open-ended question. The instrument covered several domains. The demographic section gathered information on age, gender, years of experience, qualification, and Emirate. The knowledge domain included nine items on common conditions such as upper respiratory tract infections, gastroenteritis, urinary tract infections, ENT problems, skin conditions, headaches, musculoskeletal disorders, eye problems, and infectious diseases, along with ten items on chronic conditions, including hypertension, diabetes, dyslipidemia, asthma, chronic obstructive pulmonary disease (COPD), obesity, osteoarthritis, mental health, cardiovascular disease, and hypothyroidism. Additional questions addressed pediatric and women’s health, as well as clinical guideline awareness.

All knowledge items were rated on a 5-point Likert scale ranging from 1 (not confident) to 5 (very confident). The training needs domain consisted of five items assessing preferred training modes and unmet learning areas, while the barriers section explored both personal and systemic obstacles to continuing education. Content validity was established through expert review by family medicine and medical education specialists, and a pilot test with 10 GPs (excluded from the final analysis) confirmed clarity, relevance, and usability. Minor adjustments were made based on their feedback. Cronbach’s α for knowledge of common primary healthcare conditions was 0.88, whereas for knowledge of chronic primary health conditions, Cronbach’s α was 0.91. For knowledge of child and women’s health conditions, Cronbach's α was 0.92, and for the training needs domain, it was 0.81. The full questionnaire used for data collection is provided in the appendix.

Data collection procedure

Eligible participants were invited through an official EHS email containing a secure survey link. Participation was voluntary and anonymous, with electronic consent obtained prior to accessing the questionnaire. No identifiable information was collected.

Ethical considerations

The study was approved by the Research Ethics Committee at the Ministry of Health and Prevention (MOHAP) (Approval No.: MOHAP/DXB-REC/M.M.J./No.100/2025). All procedures complied with the Declaration of Helsinki, and participant confidentiality was strictly maintained.

Statistical analysis

Data were coded and analyzed in SPSS Statistics version 25 (IBM Corp., Armonk, NY, USA). Descriptive statistics were reported as frequencies, percentages, means, and standard deviations. Knowledge scores were summed and classified using modified Bloom’s cut-offs: High (≥80%), Moderate (60-79%), and Low (<60%). Training needs were analyzed as mean Likert scores, while barriers were summarized as proportions. Inferential analyses included χ² tests and Fisher’s exact tests for categorical variables and t-tests or one-way ANOVA for continuous scores. Reliability of each domain was assessed using Cronbach’s α. Statistical significance was set at p < 0.05.

## Results

Demographics

The study included 127 participants, with a response rate of 78.9%. Of the respondents, 71 (55.9%) were male and 56 (44.1%) were female. The largest age group was 41 to 50 years, comprising 46 participants (36.2%), followed by 36 to 40 years with 32 (25.2%). In terms of professional experience, 52 respondents (40.9%) had five to 10 years of experience, 43 (33.9%) had 11 to 20 years, 20 (15.7%) had less than five years, and 12 (9.4%) had more than 20 years. Most participants held an MBBS degree (115, 90.6%), while 11 (8.7%) had a diploma in family medicine. The highest proportion of respondents practiced in Sharjah (38, 29.9%), followed by Fujairah (25, 19.7%) (Table 1).

**Table 1:** Demographic Characteristics of the Participants (n=127)

Variable	Category	Frequency	Percent
Gender	Male	71	55.9
	Female	56	44.1
Age	≤ 30 years	10	7.9
	31 - 35 years	27	21.3
	36 - 40 years	32	25.2
	41 - 50 years	46	36.2
	51 years and above	12	9.4
Years of clinical experience	< 5 years	20	15.7
	5 - 10 years	52	40.9
	11 - 20 years	43	33.9
	More than 20 years	12	9.4
Highest Medical Qualification	MBBS	115	90.6
	Diploma in Family Medicine	11	8.7
	Master’s in clinical research	1	0.8
Workplace Emirate	Dubai	17	13.4
	Fujairah	25	19.7
	Sharjah	38	29.9
	Ajman	10	7.9
	Ras Al Khaimah	22	17.3
	Umm Al Quwain	15	11.8

Knowledge of common primary healthcare conditions

The majority demonstrated a high level of knowledge about common primary healthcare conditions (n=110, 86.6%), whereas 13.4% (n=17) had a moderate knowledge level. By gender, 64 males (90.1%) and 46 females (82.1%) exhibited high knowledge, with no statistically significant difference between groups (p = 0.189). Across age groups, high knowledge was most prevalent among participants aged 51 years and above (12, 100%), followed by those aged 41 to 50 years (41, 89.1%) and 31 to 35 years (23, 85.2%), with no significant association between age and knowledge level (p = 0.323). Regarding experience, all participants with more than 20 years of experience (12, 100%) demonstrated high knowledge, followed by those with five to 10 years (46, 88.5%) and 11 to 20 years (36, 83.7%), again showing no significant difference (p = 0.381). None of the associations between knowledge level and demographic characteristics were statistically significant (Table 2).

**Table 2:** Knowledge of Common Primary Healthcare Conditions (n=127)

Category (variable)	Subcategory	High Knowledge n (%)	Moderate Knowledge n (%)	p-value	χ²
Gender	Male	64 (90.1%)	7 (9.9%)	0.18	1.72
	Female	46 (82.1%)	10 (17.9%)		
Age	≤ 30 years	7 (70.0%)	3 (30.0%)	0.32	4.67
	31 - 35 years	23 (85.2%)	4 (14.8%)		
	36 - 40 years	27 (84.4%)	5 (15.6%)		
	41 - 50 years	41 (89.1%)	5 (10.9%)		
	51 years and above	12 (100.0%)	0 (0.0%)		
Years of Clinical Experience	< 5 years	16 (80.0%)	4 (20.0%)	0.38	3.07
	5 - 10 years	46 (88.5%)	6 (11.5%)		
	11 - 20 years	36 (83.7%)	7 (16.3%)		
	More than 20 years	12 (100.0%)	0 (0.0%)		
Highest Medical Qualification	MBBS	98 (85.2%)	17 (14.8%)	0.35	2.04
	Diploma in Family Medicine	11 (100.0%)	0 (0.0%)		
	Master’s in Clinical Research	1 (100.0%)	0 (0.0%)		
Workplace Emirate	Dubai	16 (94.1%)	1 (5.9%)	0.53	4.11
	Fujairah	23 (92.0%)	2 (8.0%)		
	Sharjah	33 (86.8%)	5 (13.2%)		
	Ajman	8 (80.0%)	2 (20.0%)		
	Ras Al Khaimah	19 (86.4%)	3 (13.6%)		
	Umm Al Quwain	11 (73.3%)	4 (26.7%)		

Knowledge of chronic primary health care conditions

Regarding knowledge of chronic primary healthcare conditions, most participants demonstrated high (n=81, 63.8%) or moderate knowledge (n=42, 33.1%), with few showing low knowledge (n=4, 3.1%). By gender, the difference was not statistically significant (p = 0.147). Across age groups, high knowledge was most frequent among participants aged 51 years and above (11, 91.7%) and those aged 41 to 50 years (34, 73.9%), while the lowest proportion was seen in the 31-to-35-year group (11, 40.7%). The association between age and knowledge level was statistically significant (p = 0.013). With regard to years of experience, all participants with more than 20 years of experience (12, 100%) had high knowledge, followed by those with 11 to 20 years (30, 69.8%). The lowest proportion of high knowledge was seen among those with five to 10 years of experience (28, 53.8%), showing a significant association between experience and knowledge level (p = 0.021). By qualification, the difference was not statistically significant (p = 0.523). Across emirates, the highest proportion of high knowledge was observed in Dubai (13, 76.5%), followed by Fujairah (18, 72.0%) and Sharjah (27, 71.1%) (p < 0.001) (Table 3).

**Table 3:** Knowledge of Chronic Primary Health Care Conditions (n=127)

Category (variable)	Subcategory	High Knowledge n (%)	Moderate Knowledge n (%)	Low Knowledge n (%)	p-value	χ²
Gender	Male	50 (70.4%)	20 (28.2%)	1 (1.4%)	0.14	3.83
	Female	31 (55.4%)	22 (39.3%)	3 (5.4%)		
Age	≤ 30 years	8 (80.0%)	2 (20.0%)	0 (0.0%)	0.01	19.38
	31 - 35 years	11 (40.7%)	13 (48.1%)	3 (11.1%)		
	36 - 40 years	17 (53.1%)	14 (43.8%)	1 (3.1%)		
	41 - 50 years	34 (73.9%)	12 (26.1%)	0 (0.0%)		
	51 years and above	11 (91.7%)	1 (8.3%)	0 (0.0%)		
Years of Clinical Experience	< 5 years	11 (55.0%)	9 (45.0%)	0 (0.0%)	0.02	14.91
	5 - 10 years	28 (53.8%)	20 (38.5%)	4 (7.7%)		
	11 - 20 years	30 (69.8%)	13 (30.2%)	0 (0.0%)		
	More than 20 years	12 (100.0%)	0 (0.0%)	0 (0.0%)		
Highest Medical Qualification	MBBS	75 (65.2%)	36 (31.3%)	4 (3.5%)	0.52	3.21
	Diploma in Family Medicine	5 (45.5%)	6 (54.5%)	0 (0.0%)		
	Master’s in Clinical Research	1 (100.0%)	0 (0.0%)	0 (0.0%)		
Workplace Emirate	Dubai	13 (76.5%)	4 (23.5%)	0 (0.0%)	0.00	31.74
	Fujairah	18 (72.0%)	7 (28.0%)	0 (0.0%)		
	Sharjah	27 (71.1%)	11 (28.9%)	0 (0.0%)		
	Ajman	3 (30.0%)	4 (40.0%)	3 (30.0%)		
	Ras Al Khaimah	11 (50.0%)	10 (45.5%)	1 (4.5%)		
	Umm Al Quwain	9 (60.0%)	6 (40.0%)	0 (0.0%)		

Knowledge of child and women’s health conditions

Regarding child and women’s health conditions, most participants had a high knowledge level (n=53, 41.7%), followed by moderate (n=47, 37%) and low knowledge levels (n=27, 21.3%). By gender, 27 males (38.0%) and 26 females (46.4%) had high knowledge, while 28 males (39.4%) and 19 females (33.9%) showed moderate knowledge. Low knowledge was reported in 16 males (22.5%) and 11 females (19.6%), with no statistically significant difference between genders (p = 0.635). Across age groups, the highest proportion of high knowledge was observed among participants aged 41 to 50 years (22, 47.8%), but the association between age and knowledge level was not statistically significant (p = 0.611). Regarding years of experience, no significant relationship was observed between experience and knowledge level (p = 0.467). By qualification, the differences were not statistically significant (p = 0.680). Similarly, across emirates, the association between emirate and knowledge level was not statistically significant (p = 0.259) (Table 4).

**Table 4:** Knowledge of Child and Women’s Health Conditions (n=127)

Category (variable)	Subcategory	High Knowledge n (%)	Moderate Knowledge n (%)	Low Knowledge n (%)	p-value	χ²
Gender	Male	27 (38.0%)	28 (39.4%)	16 (22.5%)	0.63	0.90
	Female	26 (46.4%)	19 (33.9%)	11 (19.6%)		
Age	≤ 30 years	3 (30.0%)	3 (30.0%)	4 (40.0%)	0.61	6.32
	31 - 35 years	10 (37.0%)	9 (33.3%)	8 (29.6%)		
	36 - 40 years	13 (40.6%)	14 (43.8%)	5 (15.6%)		
	41 - 50 years	22 (47.8%)	15 (32.6%)	9 (19.6%)		
	51 years and Above	5 (41.7%)	6 (50.0%)	1 (8.3%)		
Years of clinical experience	<5 years	6 (30.0%)	6 (30.0%)	8 (40.0%)	0.46	5.61
	5–10 years	24 (46.2%)	18 (34.6%)	10 (19.2%)		
	11–20 years	18 (41.9%)	18 (41.9%)	7 (16.3%)		
	More than 20 years	5 (41.7%)	5 (41.7%)	2 (16.7%)		
Highest Medical Qualification	MBBS	46 (40.0%)	44 (38.3%)	25 (21.7%)	0.68	2.30
	Diploma in Family Medicine	6 (54.5%)	3 (27.3%)	2 (18.2%)		
	Master's in Clinical Research	1 (100.0%)	0 (0.0%)	0 (0.0%)		
Workplace Emirate	Dubai	6 (35.3%)	8 (47.1%)	3 (17.6%)	0.25	12.40
	Fujairah	15 (60.0%)	6 (24.0%)	4 (16.0%)		
	Sharjah	18 (47.4%)	15 (39.5%)	5 (13.2%)		
	Ajman	3 (30.0%)	4 (40.0%)	3 (30.0%)		
	Ras Al Khaimah	5 (22.7%)	8 (36.4%)	9 (40.9%)		
	Umm Al Quwain	6 (40.0%)	6 (40.0%)	3 (20.0%)		

Training needs

Most participants identified a moderate need for training (n=63, 49.6%), followed by a high need for training (n=55, 43.3%). Only 7.1% (n=9) of the participants reported low need for training. By gender, 29 males (40.8%) and 26 females (46.4%) expressed a high need for training, while moderate need was reported by 36 males (50.7%) and 27 females (48.2%). A smaller proportion indicated a low need for training, with six males (8.5%) and three females (5.4%). The difference between genders was not statistically significant (p = 0.709). Similarly, the relationship between age and training need was not statistically significant (p = 0.845). In terms of years of experience, those with 11 to 20 years of experience reported the greatest high training need (24, 55.8%), while participants with more than 20 years of experience had the lowest (2, 16.7%). Most respondents with over 20 years of experience reported a moderate training need (9, 75.0%). The association between experience and training need was not statistically significant (p = 0.185). Differences across qualification groups and emirates were not statistically significant (Table 5). The majority of participants (80, 63.0%) reported that the training sessions provided by EHS had significantly helped improve their clinical skills in primary care. Additionally, 39 participants (30.7%) felt the sessions helped to some extent. Only a small proportion reported limited or no benefit, with seven (5.5%) indicating they had not attended any EHS training sessions and one (0.8%) stating that the sessions did not help at all.

**Table 5:** Training Needs Identified by Participants (n=127)

Category (variable)	Subcategory	High need for training n (%)	Moderate need for training n (%)	Low need for training n (%)	p-value	χ²
Gender	Male	29 (40.8%)	36 (50.7%)	6 (8.5%)	0.70	0.68
	Female	26 (46.4%)	27 (48.2%)	3 (5.4%)		
Age	≤ 30 years	5 (50.0%)	5 (50.0%)	0 (0.0%)	0.84	4.12
	31 - 35 years	11 (40.7%)	14 (51.9%)	2 (7.4%)		
	36 - 40 years	17 (53.1%)	14 (43.8%)	1 (3.1%)		
	41 - 50 years	18 (39.1%)	23 (50.0%)	5 (10.9%)		
	51 years and above	4 (33.3%)	7 (58.3%)	1 (8.3%)		
Years of clinical experience	< 5 years	9 (45.0%)	11 (55.0%)	0 (0.0%)	0.18	8.97
	5 - 10 years	20 (38.5%)	27 (51.9%)	5 (9.6%)		
	11 - 20 years	24 (55.8%)	16 (37.2%)	3 (7.0%)		
	More than 20 years	2 (16.7%)	9 (75.0%)	1 (8.3%)		
Highest Medical Qualification	MBBS	51 (44.3%)	56 (48.7%)	8 (7.0%)	0.64	2.51
	Diploma in Family Medicine	3 (27.3%)	7 (63.6%)	1 (9.1%)		
	Master’s in Clinical Research	1 (100.0%)	0 (0.0%)	0 (0.0%)		
Workplace Emirate	Dubai	10 (58.8%)	7 (41.2%)	0 (0.0%)	0.18	13.85
	Fujairah	10 (40.0%)	11 (44.0%)	4 (16.0%)		
	Sharjah	17 (44.7%)	19 (50.0%)	2 (5.3%)		
	Ajman	5 (50.0%)	5 (50.0%)	0 (0.0%)		
	Ras Al Khaimah	10 (45.5%)	12 (54.5%)	0 (0.0%)		
	Umm Al Quwain	3 (20.0%)	9 (60.0%)	3 (20.0%)		

Challenges in primary healthcare management

The most common difficulty reported in managing medical conditions was time constraints during consultations (n=84, 66.1%), followed by patient expectations and beliefs (n=45, 35.4%) and diagnostic uncertainty due to overlapping presentations (n=35, 27.6%). In terms of confidence in screening and cancer guidelines, most respondents felt fairly confident (n=53, 41.7%) or completely confident (n=42, 33.1%), while smaller proportions were somewhat confident (n=28, 22.0%) or not confident (n=4, 3.1%). When managing chronic conditions, the main challenges included time constraints during consultations (n=68, 53.5%) and patient adherence to treatment plans (n=68, 53.5%), followed by managing multiple comorbidities (n=61, 48.0%). Regarding pediatric care, the primary barriers were time constraints (n=80, 63.0%), diagnosing conditions with nonspecific or atypical symptoms (n=47, 37.0%), and engaging or educating parents or caregivers (n=40, 31.5%). For women’s health issues, the most cited difficulties were lack of training in women’s health (n=59, 46.5%). In terms of professional development, respondents found in-person workshops (n=101, 79.5%), practical hands-on training (n=66, 52.0%), and clinical case discussions (n=65, 51.2%) to be the most helpful. Online courses and webinars were also beneficial (n=59, 46.5%), while mentoring or peer discussions were cited less frequently (n=25, 19.7%). Preferred modes of receiving future training included in-person seminars or workshops (n=107, 84.3%), online interactive webinars (n=68, 53.5%), mentorship or peer group discussions (n=46, 36.2%), and self-based learning such as journals, modules, or EHS guidelines (n=41, 32.3%).

Barriers to professional development

The most commonly reported personal barriers to pursuing further education or professional development in primary healthcare were limited time due to high patient workload (90, 70.9%) and difficulty balancing clinical duties with ongoing education (88, 69.3%). Other personal challenges included feeling overwhelmed by multiple roles such as clinician, educator, or administrator (32, 25.2%), difficulty prioritizing education alongside family or personal commitments (27, 21.3%), and lack of motivation or energy, possibly due to burnout (16, 12.6%). Regarding system-level barriers, the most frequently cited were limited availability of flexible learning schedules or formats (63, 49.6%) and limited access to relevant courses or workshops (56, 44.1%). Other reported barriers included lack of institutional support or incentives for continuing education (41, 32.3%), insufficient recognition or reward for participation in continuing education (32, 25.2%), and lack of policies or incentives encouraging ongoing learning (22, 17.3%). A small number of respondents (10, 7.9%) mentioned other unspecified system barriers (Table 6).

**Table 6:** Barriers to Professional Development (n=127)

Question	Item (short)	Frequency (n)	Percent of 127
Personal barriers when seeking further education / professional development	Difficulty balancing clinical duties with ongoing professional education	88	69.3%
	Limited time due to high patient workload	90	70.9%
	Feeling overwhelmed by multiple roles (e.g., clinician, educator, administrator)	32	25.2%
	Lack of motivation or energy (possible burnout)	16	12.6%
	Challenging to prioritize education while managing family or personal commitments	27	21.3%
System barriers when seeking further education / professional development	Limited access to relevant courses/workshops	56	44.1%
	Lack of institutional support or incentives for continuing education	41	32.3%
	Limited availability of flexible learning schedules or formats	63	49.6%
	Insufficient recognition or reward for continuing education efforts	32	25.2%
	Lack of policies or incentives for encouraging ongoing education	22	17.3%
	Others	10	7.9%

## Discussion

To the best of our knowledge, this is the first study to investigate GPs’ knowledge, needs, and barriers in primary healthcare delivery in EHS in the UAE. The findings of this study revealed that the majority of the participants had high knowledge about common, chronic, and women’s and children’s conditions. Similarly, 92.9% of the participants reported a moderate or high need for training. Although a direct comparison of the present study with previous studies is not possible, some studies have investigated knowledge of GPs on specific conditions. For example, Tadin and Dzaja reported that GPs have low knowledge of the oral health of children [14]. However, a qualitative study by Marcussen et al. investigated the perception of GPs about the management of chronically ill patients. Their findings, based on interviews from 14 GPs, revealed that they have been providing high-quality care to patients with type 2 diabetes and COPD [15]. Similarly, in another study by Yaqub et al., who investigated the knowledge of GPs regarding chronic kidney disease (CKD), it was reported that while most recognized diabetes (88.4%) and hypertension (80%) as major CKD risk factors, awareness of other causes was limited. Only 38% were familiar with using estimated glomerular filtration rate (eGFR) for CKD evaluation, and 61.6% identified CKD as a cardiovascular risk factor. Knowledge of target blood pressure goals was low [16].

A key finding of the present study was that more than 90% of participants reported moderate or high needs for additional training. Such near‐universal demand for CME is consistent with global evidence that primary‐care doctors see continuing education as essential to maintain competence. For example, a recent survey of community GPs in China found similarly high perceived training requirements, with virtually all physicians rating further education in cardiovascular and other chronic conditions as a top priority [17]. Similarly, another study from China reported that theoretical knowledge and clinical skills were the main needs that GPs identified during training [18]. Some studies have identified current training as low. For example, a survey of 353 Greek GPs found that 56.1% rated their CME in mental health as insufficient, and over half participated in related training only once every three years or less. Higher educational scores were linked to greater decisiveness and self-confidence in managing mental health cases. While 77.6% of GPs reported knowing the appropriate treatment, only 56.1% felt confident enough to initiate it without specialist referral, and 47.5% reported low to moderate self-confidence overall [19].

Kumar et al. assessed the educational needs and barriers of GPs across Asia-Pacific using mixed methods (28 interviews and 315 survey responses). Results showed a strong demand for training on chronic diseases such as diabetes, hypertension, mental illness, and skin disorders. Most physicians preferred comprehensive education covering screening to maintenance and favored live or small-group learning over online formats. While demographic differences in perceived needs were minimal, significant variations were observed in reported barriers to best practices [20]. In the present study, 63% of EHS GPs stated that recent EHS‐sponsored training had significantly improved their clinical skills. This positive appraisal aligns with regional studies showing that most primary‐care doctors find accredited CME valuable. In a study from Saudi Arabia, 83.9% of participants reported that prior CME had been useful, with 68.9% citing improved knowledge and 47.6% citing improved skills [21].

However, our respondents also identified multiple barriers to professional development, including time constraints, workload balance, patient factors, and resource limitations. These are consistent with previous studies. For instance, a recent survey of Gulf region primary‐care physicians found that “activities held during working hours” (65% of respondents) and heavy work commitments (38%) were the most common barriers to CPD participation [22]. Similarly, European studies of guideline adherence note that physicians frequently point to time pressure and organizational constraints as obstacles [22]. EHS GPs expressed clear preferences for how they want to learn. Large majorities favored interactive, face-to-face formats: 79.5% preferred in-person workshops and 84.3% preferred seminars. This mirrors the global trend that physicians value live learning opportunities. For example, Mueller et al. investigated physicians’ preferences regarding mode of training. Their findings showed that satisfaction was higher among in-person participants, with 91% indicating they would choose the same mode again compared to 65% of virtual attendees (P < .001). In-person attendees primarily valued travel opportunities (66%) and networking (25%), while virtual attendees cited convenience (46%) and reduced travel costs (34%) [23].

Importantly, these findings should be interpreted in the context of UAE Vision 2031, which emphasizes digital transformation, innovation in healthcare, and lifelong professional development. The UAE government aims to leverage digital platforms to enhance healthcare quality, including through e-learning and virtual CME programs [24]. While in-person CME remains highly valued, integrating digital platforms could help overcome barriers such as time constraints and heavy workloads, allowing flexible, on-demand learning that aligns with national strategies. Digital CME can also facilitate tracking of learning outcomes, support standardized knowledge updates, and expand access to underserved regions, complementing traditional interactive approaches [24].

The main strength of the study is a large, multi-site sample of GPs working under EHS, which provides broad insight into the status of primary-care physician education in the UAE. The survey was comprehensive, covering multiple domains, including knowledge, needs, and barriers. However, several limitations must be acknowledged. As a cross-sectional survey, the data capture a single point in time and cannot establish causality. Reliance on self-reported perceptions introduces potential bias. Participants may overstate needs or under-report knowledge gaps due to social desirability. The barriers and preferences are subjective, and recall bias might influence how doctors remember past training utility. Although the questionnaire was designed to capture multiple domains, its length may have posed a constraint for busy clinicians, particularly given that lack of time was frequently reported as a barrier. Future studies may consider shorter or more flexible survey formats to improve participation among healthcare providers with demanding clinical schedules. In addition, the barriers to professional development identified in this study were based on participants’ self-reported perceptions, including time constraints, workload balance, patient-related challenges, and resource limitations, which may not fully capture the underlying institutional or structural determinants affecting professional development opportunities. Additionally, although the sample is from across EHS clinics, it may not represent all UAE GPs. Furthermore, direct comparison of the present findings with previous studies was limited because few studies have examined GPs’ knowledge, training needs, and professional development barriers simultaneously within similar healthcare systems. The findings should also be interpreted within the specific healthcare and policy context of the UAE, particularly in relation to national healthcare initiatives, which may limit the generalizability of the results to other healthcare systems with different organizational structures or continuing professional development frameworks. Future studies could use longitudinal or mixed-method designs to validate these findings.

## Conclusions

This study revealed that a majority of GPs have high levels of knowledge regarding common medical conditions, chronic conditions, and health issues related to women and children. The findings highlight the importance of strengthening continuing professional development programs to support GPs in maintaining clinical competence and addressing evolving primary healthcare demands. In contexts where limited literature exists on the educational needs and professional development challenges of GPs in the region, this study provides useful evidence to inform the development of targeted and accessible training initiatives. Enhancing institutional support and adopting flexible learning approaches, including digital and blended training formats, may help address barriers to professional development. Future research should build on these findings using longitudinal or mixed-method designs to evaluate the impact of targeted training interventions on physician competence and primary healthcare outcomes.

## Appendices

**Table 7:** The questionnaire used in the study

Question No.	Section	Question / Item	Response Options
Q1	Demographics	Gender	Female/Male
Q2	Demographics	Age	≤30, 31-35, 36-40, 41-50, ≥51
Q3	Demographics	Years of clinical experience	<5, 5-10, 11-20, ≥ 21
Q4	Demographics	What is your highest medical qualification?	MBBS/MD, Diploma in Family Medicine, Master's in Family Medicine, Board/Fellowship in Family Medicine, Other
Q5	Demographics	Workplace Emirate	Dubai, Sharjah, Ajman, Fujairah, Umm Al Quwain, Ras Al Khaimah
Q6	Knowledge of Common Primary Healthcare Conditions	How confident are you in diagnosing and managing the following common conditions?	Not confident, somewhat confident, fairly confident, completely confident
		Upper respiratory tract infections (e.g., common cold, flu)	Same scale
		Gastroenteritis	Same scale
		Urinary tract infections	Same scale
		ENT problems (e.g., otitis media, sore throat)	Same scale
		Headache	Same scale
		Skin diseases (e.g., acne, dermatitis)	Same scale
		Musculoskeletal disorders (e.g., fracture, sprain, back pain)	Same scale
		Eye problems (e.g., conjunctivitis)	Same scale
		Infectious diseases (e.g., COVID-19, influenza)	Same scale
Q7	Barriers – Acute Care	In your opinion, what are the most challenging aspects of managing the above conditions in primary healthcare? (Select all that apply)	Lack of training, limited access to diagnostic tools, time constraints during consultations, patient expectations and beliefs, diagnostic uncertainty, patient non-compliance, other
Q8	Preventive Care	How confident are you in your knowledge and application of current guidelines for periodic health screening and cancer prevention?	Not confident, somewhat confident, fairly confident, completely confident
Q9	Knowledge of Chronic Primary Healthcare Conditions	How confident are you in diagnosing and managing the following common chronic conditions?	Not confident, somewhat confident, fairly confident, completely confident
		Hypertension	Same scale
		Type 2 diabetes mellitus	Same scale
		Dyslipidemia	Same scale
		Asthma	Same scale
		Chronic obstructive pulmonary disease	Same scale
		Obesity	Same scale
		Osteoarthritis	Same scale
		Mental disorders (e.g., depression, anxiety)	Same scale
		Cardiovascular diseases (e.g., ischemic heart disease, heart failure)	Same scale
		Endocrine disorders (e.g., Hypothyroidism)	Same scale
Q10	Barriers - Chronic Care	In your opinion, what are the most challenging aspects of managing chronic conditions in primary healthcare? (Select all that apply)	Lack of training, Keeping up with evolving clinical guidelines, Time constraints during consultations, Patient adherence to treatment plans, Unawareness of diagnostic tools and resources, Managing multiple comorbidities, other
Q11	Knowledge – Pediatric Health Conditions	How confident are you in diagnosing and managing common pediatric health conditions?	Not confident, slightly confident, somewhat confident, fairly confident, completely confident
		Common pediatric complaints	Same scale
		Vaccination schedules and immunization-related concerns	Same scale
		Growth and development milestones	Same scale
		Adolescent care	Same scale
Q12	Barriers – Pediatric Care	In your opinion, what are the most challenging aspects of managing pediatric patients? (Select all that apply)	Communicating effectively with young children, Engaging and educating parents or caregivers, Time constraints during consultations, Medication dosing and safety in children, Diagnosing conditions with non-specifics or atypical symptoms, Addressing behavioral or developmental concerns, Other
Q13	Knowledge – Women’s Health	How confident are you in managing the following aspects of women’s health?	Not confident, slightly confident, somewhat confident, fairly confident, completely confident
		Family planning and contraceptive methods	Same scale
		Pregnancy-related care (preconception, antenatal, and postnatal care)	Same scale
		Menstrual disorders (e.g., PCOS, irregular cycles, menorrhagia)	Same scale
		Menopause (initial assessment)	Same scale
		Vaginal discharge	Same scale
Q14	Barriers – Women’s Health	In your opinion, what are the most challenging aspects of managing women's health issues? (Select all that apply)	Lack of training in women's health issues, Lack of experience in women's health issues, Lack of interest in women's health issues, Time constraints during consultations, Lack of procedural training (e.g., performing Pap smears), Cultural or social factors, Keeping up-to-date with evolving women's health guidelines and recommendations, Other
Q15	Training Needs	Have the previous training sessions provided by Emirates Health Services (EHS) helped improve your clinical skills in primary care?	Yes, significantly, Yes, to some extent, No, not at all, I have not attended any EHS training sessions
Q16	Training Methods	Please specify the types of training initiatives you found most helpful: (Select all that apply)	In-person workshops, Online courses/webinars, Clinical case discussions, Mentoring or peer discussions, Practical hands-on training, Other
Q17	Training Preferences	How do you prefer to receive training on new treatment protocols or clinical practice guidelines? (Select all that apply)	In-person seminars/workshops, Online interactive webinars, Self-based learning (e.g., journals, modules, or EHS guidelines), Mentorship or peer group discussions, Other
Q18	System Use	What are the challenges or barriers when using the knowledge management system or e-library? (Select all that apply)	I find the Knowledge Management System easy to use, I am not fully aware of the content available, I experience difficulties in accessing the platform, I have limited time to explore or use the system, I am unfamiliar with how to navigate or use the system effectively, I prefer using other sources, I do not use it at all, Other
Q19	Training Needs	In which areas do you need more training? (Please rate the following areas based on your current needs. (1 = No need for training, 4 = High need for training)	no need for training, slight need for training, somewhat need for training, high need of training
		Common chronic disease (hypertension, diabetes, dyslipidemia)	Same scale
		Mental health management (depression, anxiety)	Same scale
		Asthma management	Same scale
		Women's health	Same scale
		Pediatric health	Same scale
		Managing emergency cases	Same scale
		Preventive care and health promotion	Same scale
		Adolescent health	Same scale
Q20	Professional Development - Personal	What are the personal barriers you face when seeking further education or professional development in primary healthcare? (Select all that apply)	Difficulty balancing clinical duties with ongoing professional education, Limited time due to high patient workload, Feeling overwhelmed by multiple roles (e.g., clinician, educator, administrator), Lack of motivation or energy possibly due to burnout, Challenging prioritizing education while managing family or personal commitments, Other
Q21	Professional Development - System	What are the main system barriers you face when seeking further education or professional development in primary healthcare? (Select all that apply)	Limited access to relevant courses/workshops, Lack of institutional support or incentive for continuing education, Limited availability of flexible learning schedules or formats, Insufficient recognition or reward for continuing education efforts, Lack of policies or incentives for encouraging ongoing education, Other
Q22	Additional Feedback	Please share any conditions, system-level issues, or patient population trends that you believe are underrepresented in current training or practice.	Open-ended response
